# Supplementary material for: Immunohistochemical Profiling of IDO1 and IL4I1 in Head and Neck Squamous Cell Carcinoma: Interplay for Metabolic Reprogramming?
Source: Int J Mol Sci. 2025 Apr 15;26(8):3719. doi: 10.3390/ijms26083719 (PMC12028074; doi:10.3390/ijms26083719)
Supplement: Supplementary file 1 [file ijms-26-03719-s001.zip › ijms-3496889-supplementary.pdf]

Supplementary Material

Immunohistochemical Profiling of IDO1 and IL4I1 in Head and Neck Squamous Cell Carcinoma: Interplay for Metabolic Reprogramming?

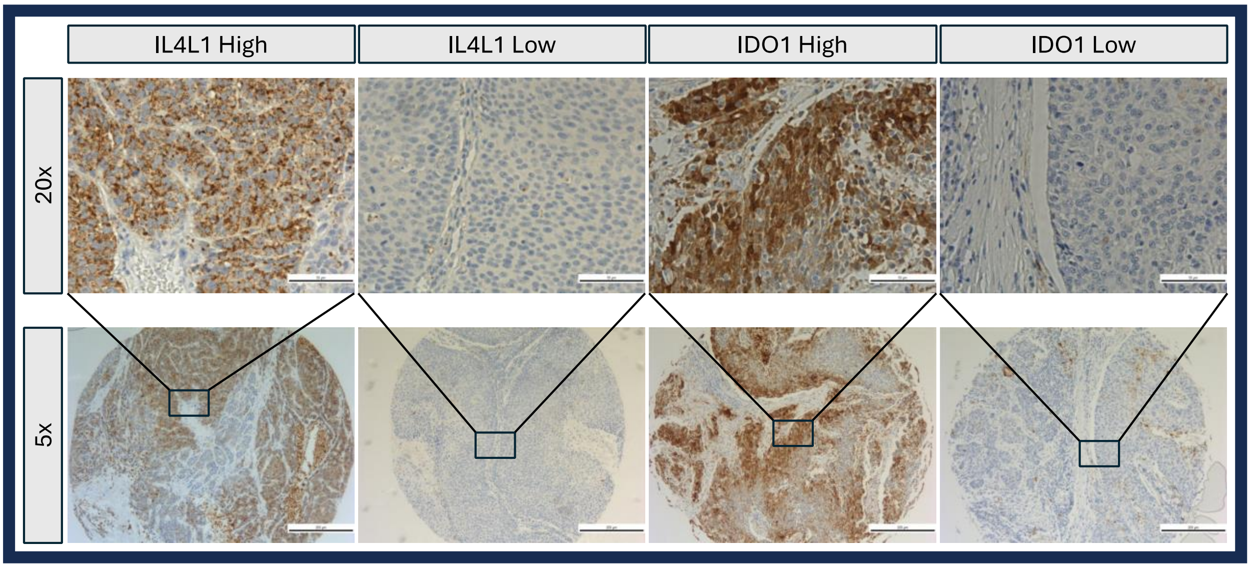

Figure S1. Representative immunohistochemical images of IL4I1 and IDO1 expression in the HNSCC tissue microarray (TMA). The lower panel (5× magnification) shows whole TMA cores with selected regions of interest outlined. The upper panel (20× magnification) presents higher-magnification images of these regions to highlight staining intensity and cellular localization. Scale bars: 200 µm (5× images) and 50 µm (20× images).

	Cutoff	Low Expression (n, %)	High Expression (n, %)	Mean Expression (%)	Standard Deviation
IDO1	14%	68 (19.1%)	288 (80.9%)	10.5%	8.5%
IL4I1	0%	236 (66.3 %)	120 (33.7%)	2.3%	3.0%

Figure S2. Distribution of low and high expression for IDO1 and IL4I1 with cutoff values determined using the maxstat method. The cutoffs were used to separate high and low expression groups for the subsequent survival analysis.
